# Evaluating cholesterol de novo synthesis biomarkers: a systematic review and meta-analysis of cancer prognosis and clinical outcomes

**DOI:** 10.1186/s12885-025-14633-8

**Published:** 2025-07-24

**Authors:** Eman Taha Osman Ali, Nouh Saad Mohamed, Emmanuel Edwar Siddig, Mai Abdul Rahman Mohammed Masri

**Affiliations:** 1 Department of Histopathology and Cytology, University of Khartoum, Khartoum, Sudan; 2 College of Medicine, Ewha Womans University, Seoul, 03760 Republic of South Korea; 3 Sirius Training and Research Centre, Khartoum, Sudan; 4 Faculty of Medical Laboratory sciences, University of Khartoum, Khartoum, Sudan; 5 Pan Africa Biomedical Institute, Kigali, Rwanda; 6 Molecular Biology Department, Faculty of Zoology, University of Khartoum, Khartoum, Sudan

**Keywords:** Cholesterol synthesis, Prognostic markers, Metabolic reprogramming, Solid tumors

## Abstract

**Background:**

While systemic cholesterol levels are generally associated with cancer risk and progression in various tumors, studies of cholesterol de novo synthesis by cancer cells in various tumor settings were limited. This meta-analysis aims to provide a comprehensive understanding of the role of cholesterol de novo synthesis pathway in cancer, focusing on key markers related with this metabolic reprogramming in cancer tissues.

**Methods:**

A systematic review and meta-analysis were conducted using data from multiple databases, including PubMed, EMBASE, and Cochrane Library. Studies were included if they examined the expression of cholesterol synthesis markers in solid tumors and reported hazard ratios (HRs) for overall survival (OS), disease-free survival (DFS), or recurrence-free survival (RFS). Data extraction and quality assessment were performed by two independent researchers. Pooled HRs and odds ratios (ORs) with 95% confidence intervals (CIs) were calculated using random-effects models.

**Results:**

Twenty studies involving 4,343 patients were included. High expression of cholesterol metabolism and esterification markers was significantly associated with worse prognosis in overall survival (OS: HR 2.38, 95% CI 1.97–2.87, *p* < 0.0001) and disease-free survival (DFS: HR 2.44, 95% CI 1.69–3.51, *p* < 0.0001). However, no significant association was observed for recurrence-free survival (RFS: HR 0.95, 95% CI 0.28–3.24, *p* = 0.9), with substantial heterogeneity (I² = 89%). Elevated expressions of enzymes correlated with more aggressive tumor characteristics, including lymph node metastasis and larger tumor size.

**Conclusions:**

High expression of cholesterol metabolism markers in solid tumors is linked to poorer survival and aggressive disease features. Among these, SQLE and SOAT1 stand out as the most robust predictors and potential therapeutic targets, emphasizing the critical role of cholesterol metabolic reprogramming in cancer progression.

**Supplementary Information:**

The online version contains supplementary material available at 10.1186/s12885-025-14633-8.

## Background

Recent studies have increasingly identified metabolic reprogramming as a hallmark of cancer [1–4]. In this context, enzymes involved in macromolecule synthesis and their transcription factors help elicit an individual pathway risk for future outcomes. These biochemical markers aid in developing personalized treatments and understanding cancer growth and metabolic shifts [5–8]. Among metabolic dysregulated pathways, cholesterol synthesis in cancer cells has been linked to cancer progression and poor outcomes [9, 10].

Cholesterol, initially recognized for its structural role in the plasma membrane, is now understood to be essential for maintaining membrane fluidity and mediating signal transduction pathways [10, 11]. In normal cells, cholesterol is primarily obtained through uptake from circulating lipoproteins and intestinal absorption, with the liver playing a central role in its systemic regulation [12, 13]. In contrast, tumor tissues often undergo metabolic reprogramming to favor de novo cholesterol synthesis [9, 10, 14], which not only supports membrane integrity but also contributes to tumor initiation, invasion, metastasis, and maintenance of cancer stem cells [15–18]. Notably, enhanced cholesterol metabolism in drug-resistant cancers promotes chemoresistance by facilitating drug efflux, supporting survival signaling within lipid rafts, and inhibiting apoptosis, thereby reducing the efficacy of anticancer therapies [19]. Additionally, elevated cholesterol levels within tumors lead to increased uptake by infiltrating CD8^+^ T cells, resulting in T-cell exhaustion and impaired cytotoxic activity, which diminishes antitumor immunity and contributes to immune evasion [20, 21]. Despite growing evidence linking cholesterol metabolism to cancer progression and therapy resistance, the precise mechanisms of de novo cholesterol reprogramming in cancer remain unclear, highlighting the need for further research into cholesterol-related pathways and biomarkers across different cancer types.

The regulation of cholesterol synthesis is primarily controlled by sterol regulatory element-binding proteins (SREBPs), which are transcription factors that activate the expression of genes involved in cholesterol and fatty acid biosynthesis [22, 23]. SREBP-1, in particular, has been implicated in the regulation of cholesterol metabolism in cancer cells [24–28]. Additionally, enzymes such as squalene epoxidase (SQLE) and sterol O-acyltransferase 1 (SOAT1) play significant roles in cholesterol biosynthesis and esterification, respectively, and their dysregulation has been associated with cancer progression [29–33].

This meta-analysis consolidates recent findings on the role of cholesterol de novo synthesis biomarkers in cancer prognosis and clinical outcomes. While our analysis provides valuable insights into this metabolic pathway, several gaps persist in understanding the full scope of cholesterol biosynthesis’s involvement in cancer. Further research is needed to elucidate the specific contributions of cholesterol synthesis-related genes, their interactions with other metabolic pathways, and their influence on tumor immunity and the tumor microenvironment. Addressing these gaps will enhance our comprehension of cholesterol de novo synthesis in cancer and inform the development of targeted therapeutic strategies.

## Methods

### Study design and search strategy

This meta-analysis was conducted following the PRISMA (Preferred Reporting Items for Systematic Reviews and Meta-Analyses) guidelines to ensure transparency and reproducibility [34]. A completed PRISMA checklist was provided in Supplementary Table 4 (Supplementary File 2). Meta-analysis was conducted using the meta package in R to evaluate the prognostic significance of cholesterol synthesis markers in solid tumors. Initially, we conducted a comprehensive systematic search of core databases (PubMed, EMBASE, Cochrane Library) up to March 31, 2024. Subsequent searches in additional databases (Web of Science, ScienceDirect Biomedical, Google Scholar) were performed within the same period, yielding no new eligible articles. Keywords for the search included; (Sterol Regulatory Element-Binding Protein-2 OR SREBP2,Sterol Regulatory Element-Binding Protein 1 OR SREBP1, Sterol O-Acyltransferase 1 OR SOAT1, OR 3-Hydroxy-3-Methylglutaryl-CoA Reductase OR HMGCR OR SERBPs OR Sterol Regulatory Element-Binding Proteins, OR Cholesterol synthesis AND (cancer OR tumor OR tumor OR carcinoma OR adenocarcinoma OR neoplasia OR neoplasm) AND (prognosis OR prognostic OR prognoses OR survival OR outcome).By incorporating these methods and keywords, the study aims to provide a comprehensive evaluation of the prognostic value of cholesterol synthesis markers in solid tumor.

### Inclusion and exclusion criteria

Studies were included if they met the following criteria: Examined the expression of cholesterol synthesis markers in solid tumors, reported hazard ratios (HRs) for overall survival (OS), disease-free survival (DFS), or recurrence-free survival (RFS), provided enough information to calculate odds ratios (ORs) for clinicopathological features such as lymph node metastasis (LNM), tumor size, and differentiation, published in peer-reviewed journals with full-text available in English.

Studies were excluded if they: Focused on non-solid tumors, lacked sufficient data to calculate HRs or ORs, were reviews, editorials, or conference abstracts without original data.

### Data extraction and quality assessment

Two independent researchers extracted data and assessed the quality of the included studies using established criteria. Discrepancies were resolved through a third independent reviewer to adjudicate any remaining disagreements. Extracted data included study characteristics, patient demographics, marker expression levels, and clinical outcomes. This meta-analysis was not registered prior to this submission.

For these cohort studies, the methodology was assessed based on a set of predefined criteria [35, 36]. These criteria were used to evaluate key methodological aspects of each study, including study design, inclusion/exclusion criteria, blinding, and statistical analysis. A maximum score of 9 was assigned based on the Newcastle-Ottawa Scale (NOS) [37].

### Methodological quality and evidence certainty

GRADE (Grading of Recommendations, Assessment, Development and Evaluation) approach was used to evaluate the certainty of evidence for each pooled outcome. GRADE considers factors such as risk of bias, inconsistency, imprecision, and publication bias to categorize the overall certainty of evidence as high, moderate, low, or very low [38]. To assess the methodological rigor of the systematic review, the AMSTAR 2 (A MeaSurement Tool to Assess systematic Reviews) checklist was applied. This tool evaluates critical domains including protocol registration, comprehensiveness of the literature search, justification for exclusions, risk of bias assessment, and the appropriateness of meta-analytical methods. AMSTAR 2 consists of 16 items, 7 of which are critical. Each item was rated as ‘yes,’ partly yes,’ or ‘no’ based on the degree to which the assessment criteria were met [39].

### Clinicopathological variables categorization and analysis

In this analysis, clinicopathological variables were categorized based on standard thresholds reported in the included studies. Binary comparisons between groups (e.g., high vs. low expression, small vs. large tumors), the calculation of odds ratios (ORs) for meta-analysis, variables were categorized according to commonly used clinical or study-specific thresholds. Associations between variables were assessed using the Chi-square test, and ORs were subsequently calculated to quantify effect sizes. Age was typically dichotomized as < 50 vs. ≥50 years or < 60 vs. ≥60 years, depending on the study cohort. Tumor size was categorized using cutoffs of 2 cm, 4 cm, or 5 cm, reflecting clinically relevant thresholds for tumor staging (small tumor size vs. large size). Tumor differentiation or grade was grouped as well/moderate vs. poor differentiation to represent differences in tumor aggressiveness.

### Statistical analysis

HRs were used to assess the impact of cholesterol synthesis markers on OS, DFS, and RFS. ORs were used to evaluate associations with clinicopathological features. Pooled HRs and ORs with 95% confidence intervals (CIs) were calculated using random-effects models for low, moderate and high heterogeneity. The Hartung-Knapp-Sidik-Jonkman (HK) method, a robust approach for random-effects meta-analysis to adjust for small sample sizes and provide more conservative estimates of the overall effect size. Heterogeneity was assessed using the I² statistic, I^2^ is measured on a scale of 0% (no heterogeneity) to 100% (high heterogeneity) and values of 25%, 50%, and 75% are considered benchmarks of low, moderate, and high heterogeneity, respectively. We performed all analyses using the “meta” package in R version 4.3.0 Foundation for Statistical Computing, Vienna, Austria).

### Publication bias

Publication bias was evaluated using Egger’s test and Begg’s test. These tests assess the asymmetry of the funnel plot to determine the presence of publication bias. Egger’s test is a regression method used to detect funnel plot asymmetry, with a P-value < 0.05 indicating significant asymmetry. Egg’s test is a rank correlation method used to detect publication bias, with a P-value < 0.05 indicating significant bias. However, it is important to note that these tests are typically more reliable when at least 10 studies are included in the meta-analysis, as recommended to ensure sufficient power and reliability of the results [40].

## Results

### Search results and study characteristics

**Fig. 1:**
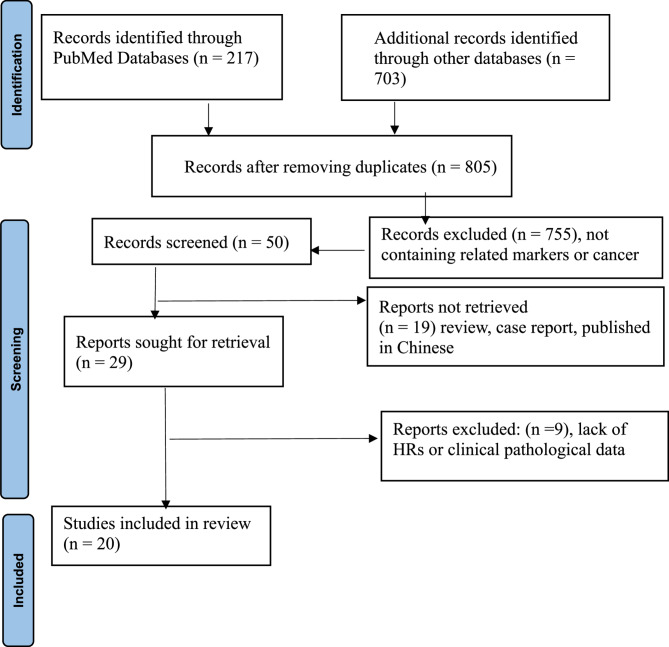
PRISMA Flowchart for selection of 20 articles. PRISMA, Preferred Reporting Items for Systematic Reviews and Meta-Analyses

The initial retrieved literature is above 920, among them 217 were filtered from PubMed, 703 were selected from other databases. After excluding duplicate and irrelevant studies that did not explore the relationship between cholesterol synthesis markers and solid tumors, 50 studies were enrolled. By browsing the study abstract, 29 studies were identified after excluding reviews, letters and case reports. After reading the complete text, 9 studies were excluded owing to a lack of clinicopathological data and enough data for HRs. Finally, 20 studies were included [41–60] in our analysis based on the predefined criteria, and the selection process is detailed in Fig. 1. Among the 20 studies, 13 were from China, 2 from South Korea, 2 Sweden and one was from Germany, Ireland, and Brazil. with sample sizes ranging from 34 to 621. Eighteen studies contributed data to the overall survival (OS) analysis. Since Hong et al. [48] reported OS outcomes for two different cancer types, the analysis included 19 effect sizes. Eight studies reported data for disease-free survival (DFS), and five studies reported recurrence-free survival (RFS). Brennan et al. [59] contributed only RFS data, and Gustbée et al. [58] contributed only DFS data. The meta-analysis included a total of 4,343 patients. The detailed Study characteristics are presented in Table 1.

**Table 1:** Main characteristics of eligible studies

Author	Year	Country	Cancer	Marker	Gender M/F	Follow up time	No. patients	IHC, Cut-off point	Survival Data
Bao	2016	China	Breast cancer	SREBP-1	0/82	60 months	82	High:>1, Low: <1	DFS/OS
Li	2014	China	Hepatocellular Carcinoma	SREBP-1	29/18	22.5 months	47	High:>1, Low: <1	DFS/OS
Zhang	2018	China	Breast cancer	SREBP-1	0/329	100 months	329	N. A	OS/DSS
Kim	2021	South. Korea	Breast cancer	SQLE	0/198	≥ 10 years	198	High: 3, Low: 0–2	DFS/OS
Zhang	2014	China	Lung cancer	SQLE	46/19	60 months	65	RT-PCR	OS
Kim	2019	South. Korea	Colorectal cancer	SQLE	75/68	65.0 months	143	High: >25%, Low:<25%	RFS/OS
Jiang	2021	China	Colon adenocarcinoma	SQLE	124/109	5 years	233	N. A	RFS/OS
Hong	2022	China	Lung cancer	SQLE	N. A	3 years	179	High:> median, Low:< median	DFS/OS
Hong	2022	China	Breast cancer	SQLE	0/422	127 months	442	High:> median, Low:< median	DFS/OS
Yang	2014	Taiwan, China	Lung cancer	SQLE	N. A	120 months	135	High: 2,3, Low: s 0, 1	DFS/OS
Li	2022	China	Head and Neck Squamous Cell	SQLE	78/37	25 months	115	N. A	PFS/OS
Liu	2024	China	Colorectal cancer	SQLE	N. A	5 years	272	N. A	DFS/OS
Chao	2023	China	Gastric cancer	SQLE	66/41	60 months	107	High: <30%, Low: ≥30%	OS
Li	2022	China	Colorectal Cancer	SQLE	117/90	5 years	207	High: >10%, Low: <10%	OS
Eckhardt	2021	Germany	Prostate cancer	SOAT1	305/0	5 years	305	High: <3 Low: >3	OS
Lacombe	2020	Brazil	Adrenocortical Carcinoma	SOAT1	33/79	180 months	112	High ≤ 2, Low > 2	RFS/PFS/OS
Wang	2022	China	Colorectal cancer	SOAT1	22/91	100 months	113	High:> median, Low:< median	DFS/OS
Zhu	2021	China	Gastric Cancer	SOAT1	N. A	5 years	160	High: 8–12, Low: 0–6	OS
Gustbée	2015	Sweden	Breast cancer	HMG-CoA reductase	0/885	11 years	885	High: 2/3, Low: 0,1	DFS
Brennan	2011	Ireland	Breast cancer	HMG-CoA reductase	0/422	5.5 years	422	High: 1–2+, Low: 0	RFS
Butt	2014	Sweden	Breast cancer	HMG-CoA reductase	0/458	13.8 years	458	High: >10%, Low: <10%	PFS/OS

*M/F *Male/Female, *No. patients* Number of patients included in the study, *IHC* Immunohistochemistry, *DFS* Disease-Free Survival, *OS* Overall Survival, *RFS* Recurrence-Free Survival, *DSS* Disease-Specific Survival, *PFS* Progression-Free Survival

### Relationship between expression of cholesterol synthesis markers and cancer survival

**Fig. 2:**
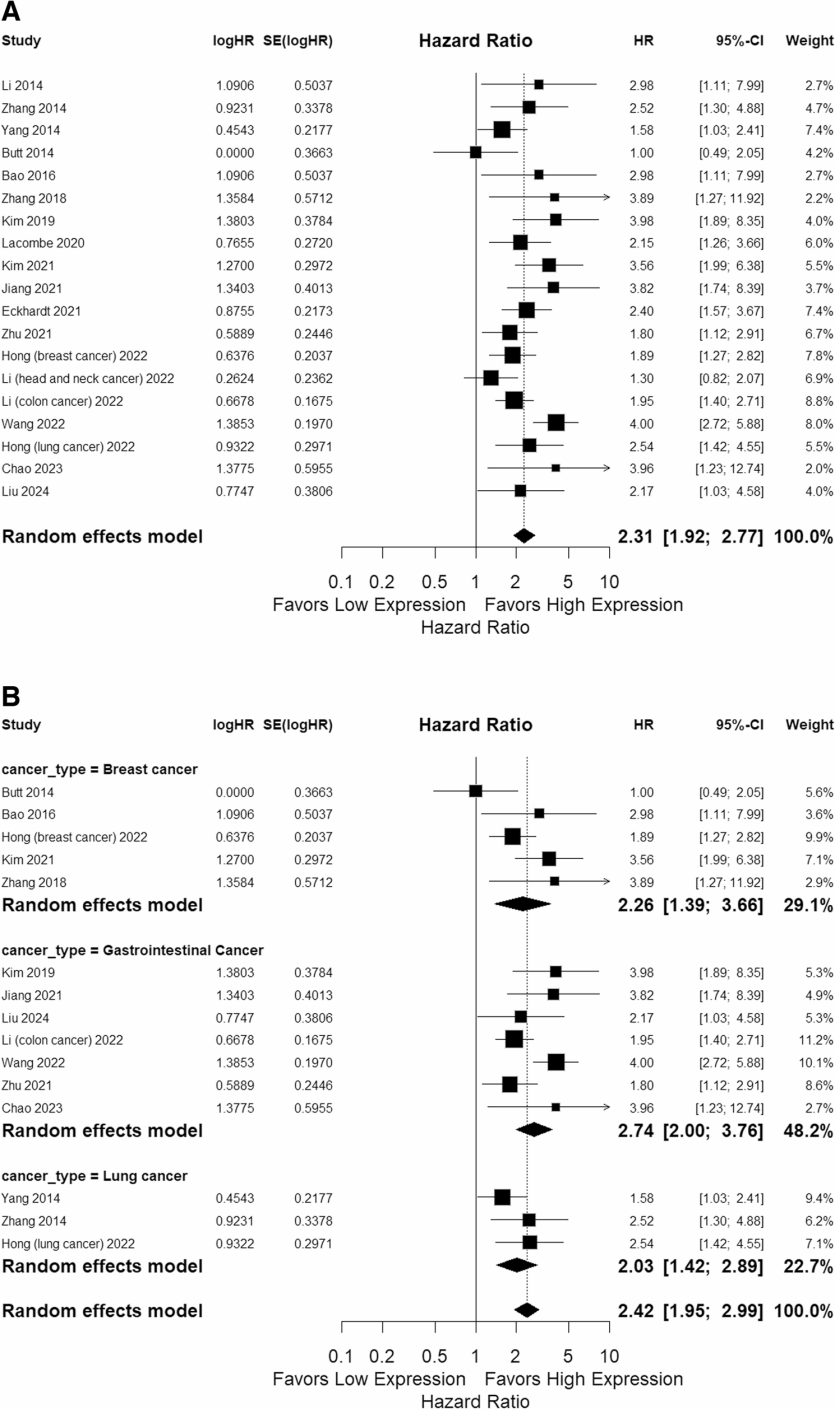
Forest plot (Random-effects model) of the hazard ratio (HR) for the association between the high expression. **A **presents the pooled HR for OS across 18 studies evaluating the effect of cholesterol synthesis markers among solid tumors, **B** shows subgroup analyses by cancer type of cholesterol synthesis markers and overall survival (OS) of patients with solid tumors

The pooled analysis of 18 studies evaluating the effect of cholesterol synthesis markers on overall survival (OS) yielded a significant hazard ratio (HR) of 2.3 (95% CI: 1.9 to 2.8, *p* < 0.0001), indicating a negative prognostic value of these markers in cancer. Heterogeneity across the studies was moderate, with an I² value of 45%. Subgroup analysis by cancer type revealed the following findings: For breast cancer (*n* = 5), the random-effects model yielded an HR of 2.3 (95% CI: 1.4 to 3.7, *p* < 0.0001) with low heterogeneity (I² = 29%); for lung cancer (*n* = 3), yielding an HR of 2 (95% CI: 1.4 to 3, *p* < 0.0001) with low heterogeneity (I² = 23)%; and for gastrointestinal cancer (*n* = 7), the random-effects model showed an HR of 2.7(95% CI: 2 to 3.7, *p* < 0.0001), with moderate heterogeneity (I² = 48%). The summary of these pooled HRs is presented in Fig. 2A and B. No significant publication bias was found for OS, with Egger’s test yielding a *p*-value of 0.21.

**Fig. 3:**
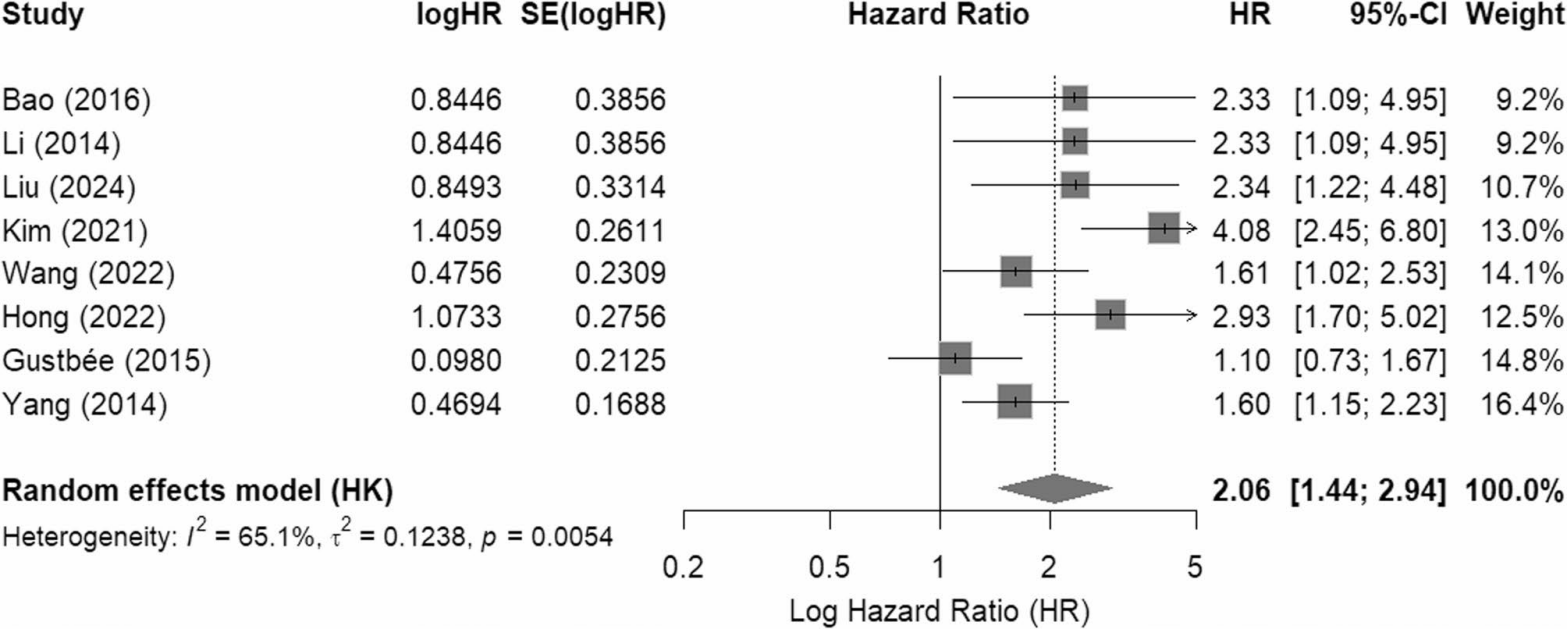
Forest plot (Random-effects model) of the hazard ratio (HR) illustrating the association between cholesterol synthesis markers and disease-free survival (DFS)

**Fig. 4:**
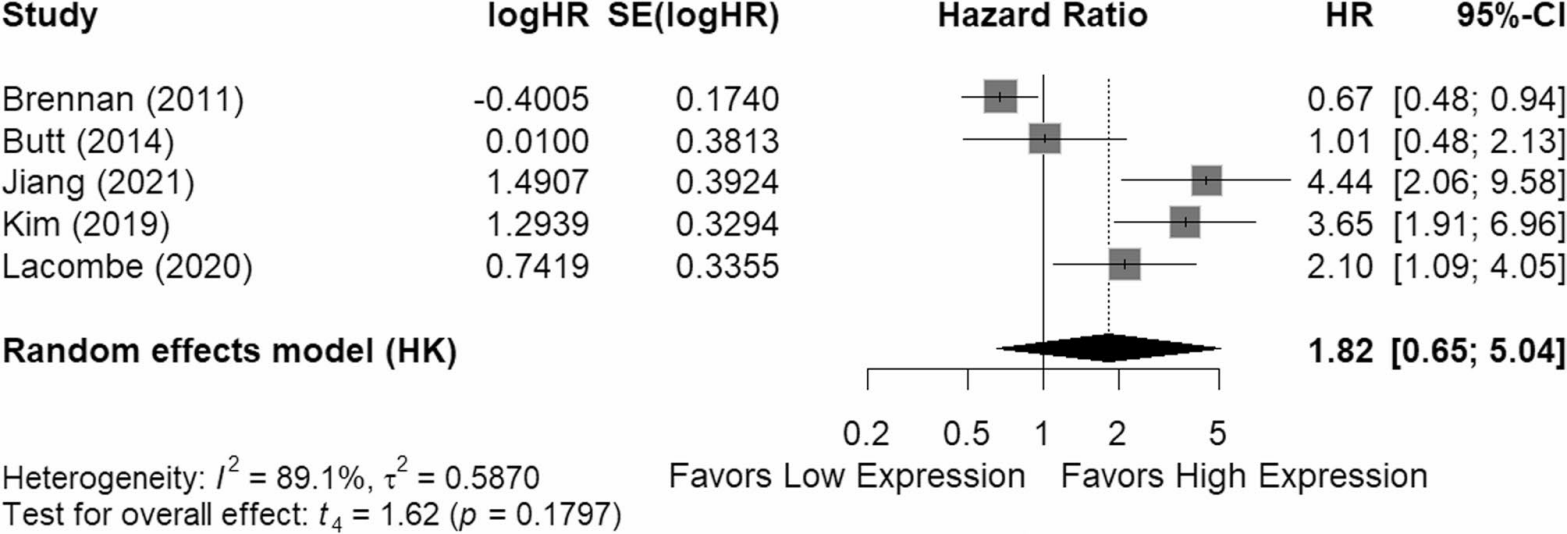
Forest plot (Random-effects model) of the hazard ratio (HR) demonstrating the relationship between cholesterol synthesis markers and recurrence-free survival (RFS)

The analysis of disease-free survival (DFS) involved 8 studies and revealed a significant association between cholesterol synthesis markers and DFS (HR = 2.0598, 95% CI: 1.4445 to 2.9371, *p* = 0.001), although high heterogeneity was observed (I² = 65%, *p* < 0.01). Publication bias was not detected, as indicated by a non-significant funnel plot asymmetry (*p* = 0.1914) from Egger’s test (Fig. 3). In contrast, the recurrence-free survival (RFS) analysis, including 5 studies, found no significant association between cholesterol synthesis markers and RFS (HR = 0.9531, 95% CI: 0.2806 to 3.2371, *p* = 0.9265), with substantial heterogeneity (I² = 89%, *p* < 0.0001) (Fig. 4). Again, no significant publication bias was found, with Egger’s test yielding a *p*-value of 0.07.

### Geographic subgroup analysis

Our meta-analysis also explored the potential impact of geographical location on the association between cholesterol synthesis markers and cancer survival. The results indicated that both the China (*n* = 13) and non-China (*n* = 7) groups showed consistent pooled hazard ratios (HRs). In the China subgroup, the pooled HR was 2.53 (95% CI: 1.89 to 3.34), while the non-China subgroup exhibited a pooled HR of 2.09 (95% CI: 1.32 to 3.31). Both subgroups demonstrated moderate to substantial heterogeneity, with I² values of 44% for the China group and 57.5% for the non-China group. This suggests that the observed variability in effect sizes is likely attributable to factors other than geographical differences. Supplementary Fig. 1A, 1B. Further analyses for RFS and DFS were not applicable due to the limited number of studies available for these outcomes.

### Subgroup analysis by cholesterol synthesis markers

Subgroup analysis by specific cholesterol synthesis markers revealed varying prognostic implications for different markers. For SREBP-1 (*n* = 3), the HR for overall survival (OS) was 3.2 (95% CI: 2.22 to 4.63, *p* = 0.0053), with no observed heterogeneity. For SQLE (*n* = 11), the HR was 2.35 (95% CI: 1.8 to 3, *p* < 0.0001), with moderate heterogeneity (I² = 39%). For SOAT1 (*n* = 4), the HR for OS was 2.5265 (95% CI: 1.4435 to 4.4223, *p* < 0.0001), with substantial heterogeneity (I² = 61%). However, it is important to note that HR data for HMGCR was available from only a single study, so no subgroup analysis was conducted for this marker. These findings suggest that the impact of cholesterol synthesis markers on OS varies across different markers, with certain markers showing stronger associations and varying levels of heterogeneity. Supplementary Fig. 2. This analysis for RFS and DFS was not applicable due to the limited number of studies available for these outcomes.

### Relationship between expression of cholesterol synthesis markers and of clinicopathological features

Regarding clinicopathological features, significant associations were found between cholesterol synthesis markers and key tumor characteristics, including lymph node status (positive vs. negative), tumor size, and tumor differentiation (poor vs. well/moderate). Specifically, the odds ratio (OR) for nodal status was 1.2798 (95% CI: 1.0863 to 1.5079, *p* = 0.0032, I² = 77.6%), for tumor size was 1.6929 (95% CI: 1.2994 to 2.2057, *p* < 0.0001, I² = 42.2%), and for tumor differentiation was 1.4516 (95% CI: 1.0807 to 1.9497, *p* = 0.0133, I² = 78.9%). These findings suggest that cholesterol synthesis markers are associated with more aggressive tumor features, including larger size, poorer differentiation, and increased likelihood of lymph node metastasis. However, no significant association was found with age (OR = 1.1071, 95% CI: 0.8154 to 1.5031, *p* = 0.5143, I² = 14%), suggesting that age does not influence the expression of cholesterol synthesis markers. Table 2 provides a detailed summary of these associations.

**Table 2:** Summary of clinicopathological features associated with cholesterol synthesis markers

Categories	OR	95% CI	*p*-value	I^2	*p*-value
Age	1.1071	0.8154; 1.5031	0.5143	14.0%	> 0.05
Differentiation (poor/well + moderate)	1.4516	1.0807; 1.9497	0.0133	78.9%	< 0.05
Nodal status (positive/negative)	1.2798	1.0863; 1.5079	0.0032	77.6%	< 0.05
Tumor size (< 5 cm)	1.6929	1.2994; 2.2057	< 0.0001	42.2%	< 0.05

### Quality assessment and sensitivity analyses

**Fig. 5:**
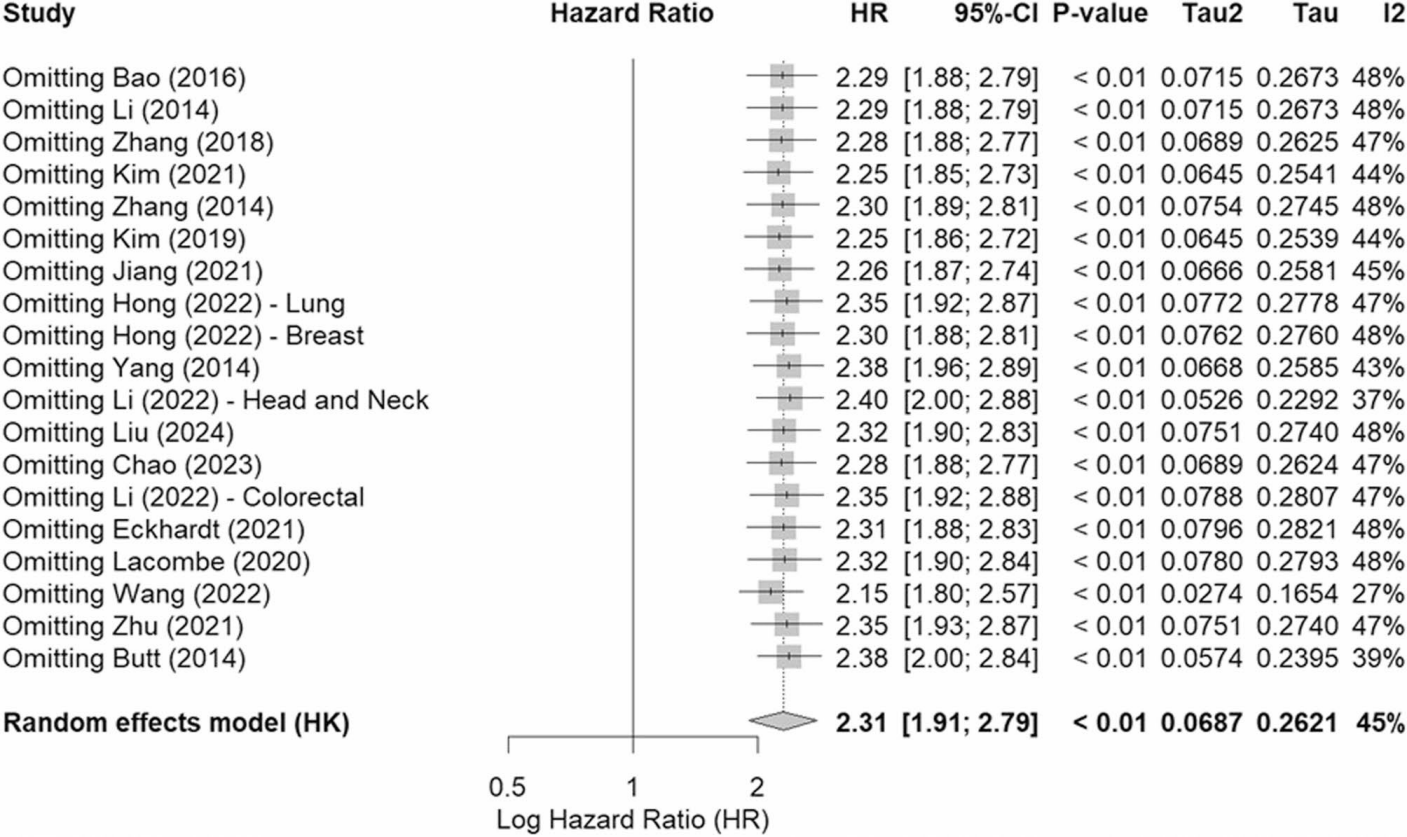
Sensitivity Analysis of Overall Survival (OS) Hazard Ratios

All the included studies were cohort studies, and each achieved a Newcastle-Ottawa Scale (NOS) score of ≥ 6, classifying them as high-quality studies. Additionally, all studies involved immunohistochemistry, and 22 key methodological characteristics essential for reporting in prognostic immunohistochemical studies were evaluated. All studies scored ≥ 15 in this assessment, further validating their high methodological quality. Supplementary Table 1.

sensitivity analysis revealed that no matter which single study was omitted, the overall pooled analysis is stable, and no individual study dominated this meta-analysis. Summary of sensitivity analysis was demonstrated in Fig. 5. Further, analysis excluding studies without cut-off values included 14 studies and yielded consistent results (HR = 2.3906, 95% CI: 1.9122 to 2.9888, *p* < 0.0001, I² = 45.1%), as shown in Supplementary Fig. 3. Further subgroup analysis was applied comparing studies using different cutoff methodologies: the high/low threshold group (*n* = 11 studies, HR = 2.45, 95% CI: 1.98–3.04) and the median-based cutoff group (*n* = 3 studies, HR = 2.31, 95% CI: 1.65–3.24). No significant difference was observed between these groups based on cutoff determination criteria (*p* > 0.05), indicating that the prognostic value of cholesterol metabolism markers remains consistent regardless of whether predetermined thresholds or data-driven median cutoffs are employed.

Leave-one-out analysis demonstrated robust results for DFS with no single study substantially altering the pooled effect, while RFS analysis was significantly influenced by Brennan [59], whose removal shifted the effect from non-significant (HR = 1.82, *p* = 0.180) to significantly harmful (HR = 2.43, 95% CI: 1.29–4.56, *p* = 0.006). Subgroup analysis revealed that SQLE consistently predicted worse outcomes for both DFS (4 studies; HR = 2.51, 95% CI: 1.64–3.86, I² = 67%) and RFS in colorectal cancer (2 studies; HR = 4.04, 95% CI: 3.01–5.42, I² = 0%), while SREBP-1 showed significant association with worse DFS (2 studies; HR = 2.33, 95% CI: 1.36–3.97, I² = 0%). By cancer type, breast cancer and colorectal cancer demonstrated significant DFS associations, whereas RFS results were mixed for breast cancer due to conflicting HMG-CoA effects (HR = 0.83, 95% CI: 0.31–2.19, I² = 62.8%) and strong harmful associations for colorectal cancer, with single-study estimates for SOAT1, HMG-CoA, hepatocellular carcinoma, lung cancer, and adrenocortical carcinoma limiting comprehensive subgroup analyses.

### Methodological rigor and certainty of evidence

The AMSTAR 2 tool was rigorously applied to evaluate the methodological quality of our systematic review and meta-analysis. Two independent researchers assessed all 16 AMSTAR 2 items, with discrepancies resolved by a third reviewer. Seven critical domains (e.g., protocol registration, comprehensive search, risk of bias assessment) were prioritized. While the review achieved a high overall rating, it was not pre-registered (critical Item 2: “no”), and Item 7 (excluded studies list) was adapted to feasibility constraints and rated as non-critical. All other critical domains (Items 3, 8, 10, 11, 13, 15) were fully met (“yes”). Non-critical items were largely satisfied, with ≤ 2 unmet criteria, supporting robust methodology. Supplementary Table 2 (Supplementary File 2). The certainty of evidence, assessed using the GRADE approach, was rated as low for OS and DFS, due to moderate heterogeneity but consistent results and low risk of bias. RFS, the certainty, was considered very low, primarily due to serious inconsistency and imprecision from the limited number of studies and high heterogeneity. Supplementary Table 3 (Supplementary File 2).

## Discussion

Although systemic cholesterol levels are established risk factors for cancer, the prognostic role of tumor-intrinsic de novo cholesterol synthesis remains understudied [61, 62]. It is crucial to distinguish between circulating cholesterol levels and the intrinsic reprogramming of cholesterol metabolism in tumor cells, which may involve upregulation of pathways or adaptive mechanisms, such as increased LDL receptor (LDLR) expression to compensate for impaired synthesis [63, 64]. Such adaptations support membrane biosynthesis and energy demand essential for tumor growth and proliferation. Understanding and evaluating biomarkers of these adaptive processes is essential for uncovering their prognostic value and therapeutic potential in cancer.

Our meta-analysis, based on 20 studies encompassing 4,343 patients with various solid tumors, revealed that elevated expression of de novo cholesterol synthesis markers, including SREBP‑1, SQLE, and SOAT1, is significantly associated with poorer overall survival (OS) and disease‑free survival (DFS). These findings suggest a pivotal role for dysregulated cholesterol metabolism in driving tumor aggressiveness and progression. Nevertheless, these results should be interpreted with caution, as the limited number of studies included and variability across tumor types may restrict their generalizability. We highlight the need for future, more comprehensive research to fill these gaps and deepen our understanding of cholesterol metabolic reprogramming in cancer pathophysiology.

DFS and RFS are related yet distinct indicators of cancer progression and treatment response. DFS measures time to any recurrence or death, while RFS specifically tracks the return of the original cancer following curative therapy [65]. In our findings, DFS analyses demonstrated consistent associations between elevated expression of cholesterol synthesis markers, SREBP‑1, SQLE, and SOAT1, and poorer outcomes, particularly in breast and colorectal cancers. Conversely, RFS results were more variable; exclusion of a single study notably shifted the pooled effect toward a significantly harmful association, highlighting sensitivity to individual data points. Notably, SQLE showed persistent detrimental effects in colorectal cancer, whereas HMG‑CoA appeared protective in breast cancer. These variations may be attributed to differences in cancer types, the specific markers measured, or the inherent distinctions between DFS and RFS as prognostic measures. However, the limited number of studies and observed heterogeneity constrain the strength of these conclusions. Overall, these findings reveal critical gaps and underscore the need for more standardized, comprehensive research to clarify the role of cholesterol synthesis biomarkers in cancer recurrence and survival, independent of treatment effects.

A stratified subgroup analysis of OS was performed across different cancer types to assess the consistency of findings within specific tumor contexts. However, this analysis was constrained by the limited number of studies available for individual cancer types, particularly breast, lung, and gastrointestinal cancers. Despite these limitations, the subgroup results were broadly consistent with the overall analysis, indicating a trend toward poorer OS associated with elevated expression of cholesterol synthesis markers. Nevertheless, the small number of studies per cancer type significantly reduced the statistical power and limited the generalizability of these findings. Therefore, there is a critical need for additional well-designed, cancer-type–specific studies with larger patient cohorts to validate these associations and to further elucidate the role of cholesterol metabolism in this survival outcomes.Moreover, single-marker stratified analysis was not significantly affected by pooled outcomes, indicating that the prognostic effects of cholesterol synthesis are similar across these tumor types for different markers.

The role of cholesterol metabolism activation in tumorigenesis has been demonstrated in some studies. Zhai et al. reported that SREBP1 promotes cancer metastasis by upregulating epithelial-mesenchymal transition in colorectal cancer [66]. Endogenous cholesterol esters have also been reported to be highly expressed in cancer tissue compared to benign counterparts [67]. Specifically, the role of SOAT1 in cholesterol esterification has been attributed to cancer progression by increasing cancer lipid droplets [68]. Despite these findings, mechanistic studies exploring how these enzymes are regulated and how the cholesterol biosynthesis pathway is reprogrammed in cancer remain to be elucidated. Understanding the molecular regulation, transcriptional control, and metabolic adaptation of this pathway is essential to identify potential therapeutic targets and improve cancer management strategies.

The endogenous cholesterol synthesis pathway was also reported to have a controversial effect. Zhao et al.‘s study illustrated that inhibiting de novo cholesterol synthesis in hepatocellular carcinoma (HCC) cells promotes tumor progression by upregulating prostaglandin E [69]. Similarly, some studies reported that targeting this pathway had no beneficial effect on cancer patients [70]. This discrepancy may add further complexity to cancer metabolism reprogramming, which may vary among tissues and during different cancer stages.

Contrary to our pooled findings, HMGCR expression correlates with favorable prognosis in colon, ovarian, and breast cancers [71–73], though insufficient data prevented subgroup analysis (only one study per outcome). While HMGCR catalyzes the rate-limiting cholesterol synthesis step, aggressive tumors appear to circumvent this control through a dual bypass mechanism: actively importing exogenous squalene and converting it to 2,3-oxidosqualene via SQLE, while simultaneously using SOAT1 to esterify cholesterol for lipid droplet storage, thereby maintaining cholesterol homeostasis independently of HMGCR regulation [74, 75]. This explains why SQLE and SOAT1 overexpression consistently predicts poor prognosis [66–68], they represent metabolic escape pathways that enable sustained tumor growth despite HMGCR constraints. These findings suggest that future research should prioritize pathway-level assessments, such as evaluating SQLE/HMGCR or SOAT1/HMGCR ratios. Such approaches may offer deeper insights into the mechanisms underlying cholesterol metabolic reprogramming.

Statins, by inhibiting HMGCR, have shown anticancer potential, but clinical outcomes are inconsistent [70]. Our analysis highlights this complexity, as HMGCR expression correlates with favorable prognosis in some cancers [59, 60]. Importantly, SOAT1 and SQLE exert distinct, individual influences on statin responsiveness [76, 77]. In statin-sensitive lung cancer cells, atorvastatin reduces intracellular cholesterol esters and downregulates SOAT1 expression, whereas resistant cells maintain both, suggesting that effective statin action requires SOAT1 suppression [76]. Similarly, in prostate cancer, SOAT1 inhibition by avasimibe suppresses tumor proliferation and metastasis via the E2F-1 signaling pathway [77]. Additionally, in hepatocellular carcinoma, SOAT1 promotes epithelial-mesenchymal transition (EMT) and contributes to tumor progression by increasing cholesterol esterification [78]. Conversely, elevated SQLE expression in head and neck squamous cell carcinoma is identified as a key driver of chemoresistance and tumorigenesis, operating through a cholesterol-dependent pathway [79]. Moreover, in pancreatic cancer, SQLE promotes tumor growth by attenuating endoplasmic reticulum stress and activating lipid raft-regulated Src/PI3K/Akt signaling pathways [80]. Collectively, these findings indicate that SOAT1 downregulation may be necessary for statin sensitivity, while SQLE upregulation may drive statin resistance. Considering SOAT1 and SQLE as singular, mechanistically relevant biomarkers could enhance patient stratification and improve statin-based therapeutic strategies.

This study underscores the critical role of tumor-intrinsic cholesterol metabolism in cancer prognosis, highlighting the prognostic significance of de novo cholesterol synthesis markers such as SREBP-1, SQLE, and SOAT1. Our meta-analysis reveals consistent associations between elevated expression of these markers and, OS and DFS across various solid tumors. However, the variability observed between DFS and RFS analyses suggests that these markers’ prognostic value may be influenced by cancer type, specific markers assessed, and the inherent differences between DFS and RFS as outcome measures. Furthermore, considering the distinct influences of SOAT1 and SQLE on statin responsiveness, these markers could serve as valuable tools for patient stratification and the development of targeted therapeutic strategies. In conclusion, while preliminary evidence suggests that tumor-intrinsic cholesterol metabolism plays a pivotal role in cancer prognosis, further exploration is essential to fully understand its implications and to translate these findings into clinical practice.

### Study limitations and future research

While this study provides valuable insights into the prognostic significance of tumor-intrinsic cholesterol metabolism markers, several limitations warrant consideration. Methodological heterogeneity across included studies, particularly in immunohistochemistry (IHC) assays, poses a significant concern. Variations in antibody selection, staining protocols, scoring methods, and cutoff values for marker expression could contribute to inconsistent results and limit cross-study comparability. Additionally, the inclusion of only English-language publications introduces potential language bias, which may have led to the exclusion of relevant data from non-English studies. Clinical heterogeneity, such as differences in treatment regimens, disease stage, and comorbid conditions, may have introduced confounding effects. Although subgroup analysis revealed consistent hazard ratios between Chinese and non-Chinese studies, this result should be interpreted cautiously due to the small number of studies from outside China and the limited geographic representation overall.

To address these limitations, future research should aim to standardize research methodologies to enhance comparability across studies. Expanding the inclusion criteria to encompass non-English-language publications and studies from diverse geographic regions will help mitigate language and geographic biases, providing a more comprehensive understanding of the role of cholesterol metabolism markers in cancer prognosis. Moreover, prospective multicenter studies with well-defined patient cohorts are essential to validate these findings and explore the potential of cholesterol metabolism markers as therapeutic targets in cancer treatment.

## Supplementary Information

Supplementary Material 1: Figure 1. Geographic Subgroup Analysis of Hazard Ratios for OS (A) the pooled HR for OS studies conducted in China, (B) a non-China region.

Supplementary Material 2: Figure 2. Subgroup Analysis by Cholesterol Synthesis Markers.

Supplementary Material 3: Figure 3. Sensitivity Analysis Excluding Studies Without Cut-off Values.

Supplementary Material 4: Table 1. Quality Assessment of Included Studies.

Supplementary Material 5: Table 2. AMSTAR 2 Assessment of Methodological Rigor. Table 3. Comprehensive Assessment of Evidence. Table 4. PRISMA Checklists.

Supplementary Material 6.

## Data Availability

No datasets were generated or analysed during the current study.
