# Supplementary figures and images for: Evaluating cholesterol de novo synthesis biomarkers: a systematic review and meta-analysis of cancer prognosis and clinical outcomes
Source: BMC Cancer. 2025 Jul 24;25:1208. doi: 10.1186/s12885-025-14633-8 (PMC12291504; doi:10.1186/s12885-025-14633-8)

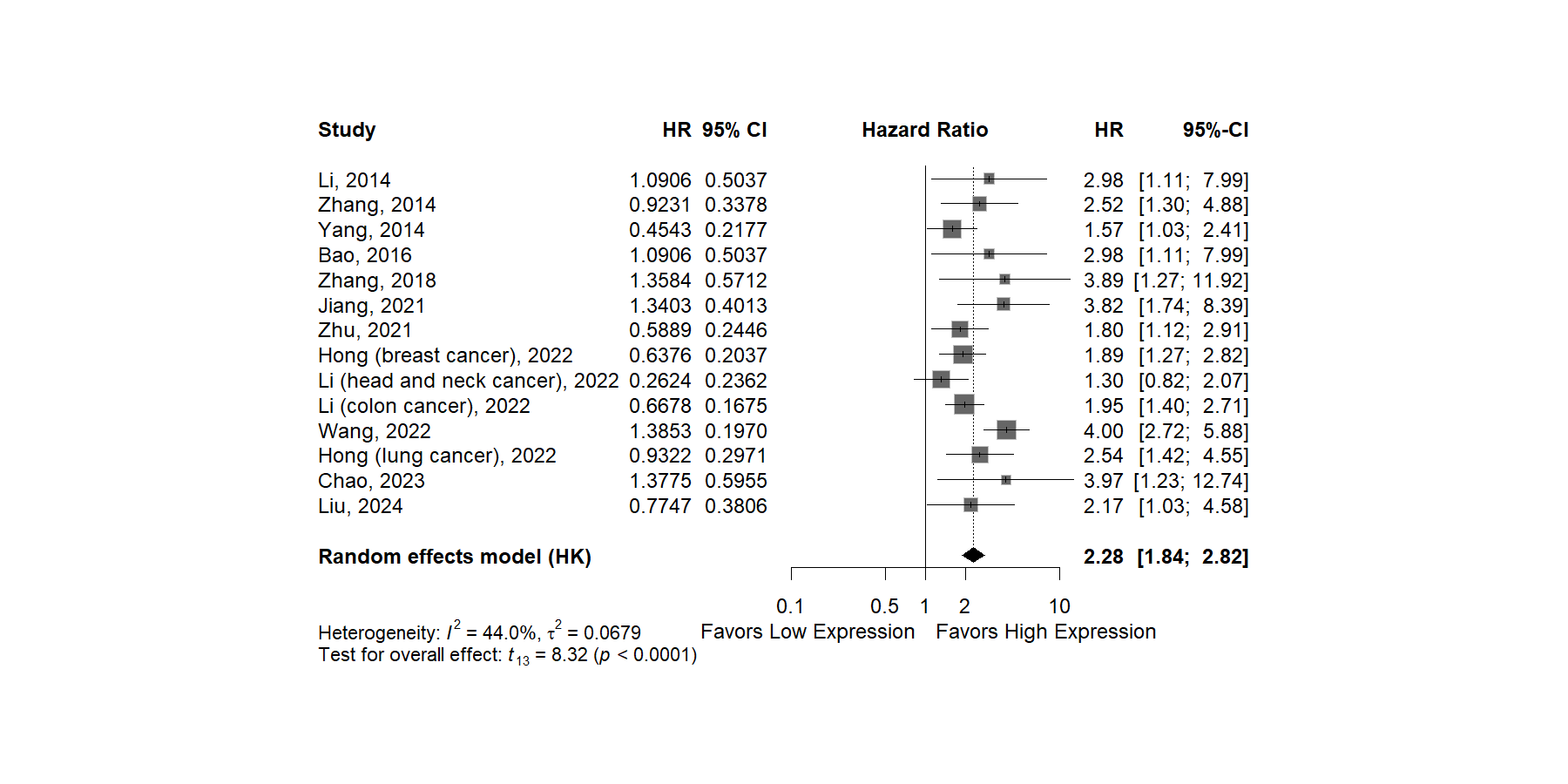

Supplement: Supplementary file 1 — Supplementary Material 1: Figure 1. Geographic Subgroup Analysis of Hazard Ratios for OS (A) the pooled HR for OS studies conducted in China, (B) a non-China region. [file 12885_2025_14633_MOESM1_ESM.zip › Fig. sup 1 A.png]

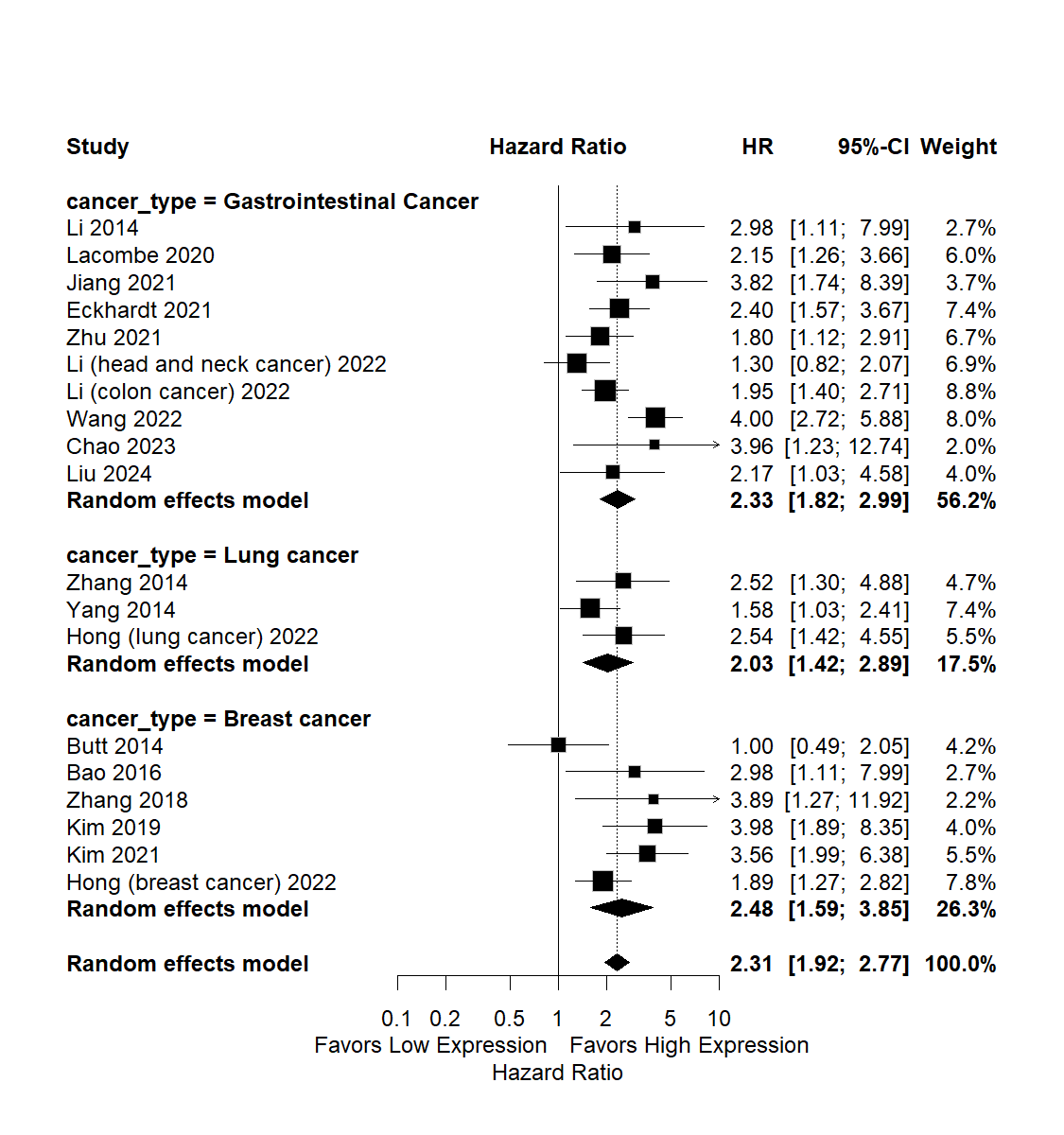

Supplement: Supplementary file 1 — Supplementary Material 1: Figure 1. Geographic Subgroup Analysis of Hazard Ratios for OS (A) the pooled HR for OS studies conducted in China, (B) a non-China region. [file 12885_2025_14633_MOESM1_ESM.zip › Fig.sup1 B..png]

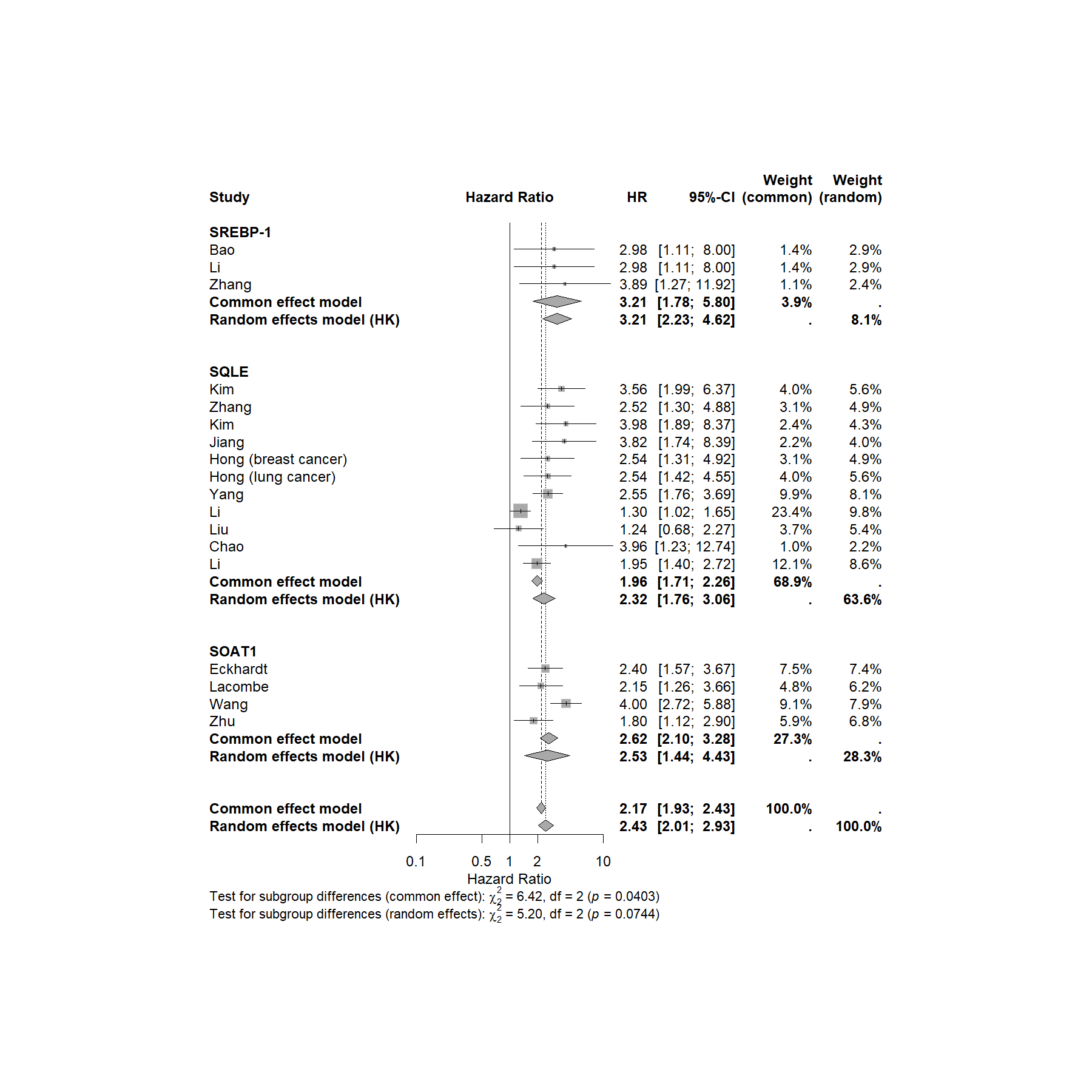

Supplement: Supplementary file 2 — Supplementary Material 2: Figure 2. Subgroup Analysis by Cholesterol Synthesis Markers. [file 12885_2025_14633_MOESM2_ESM.png]

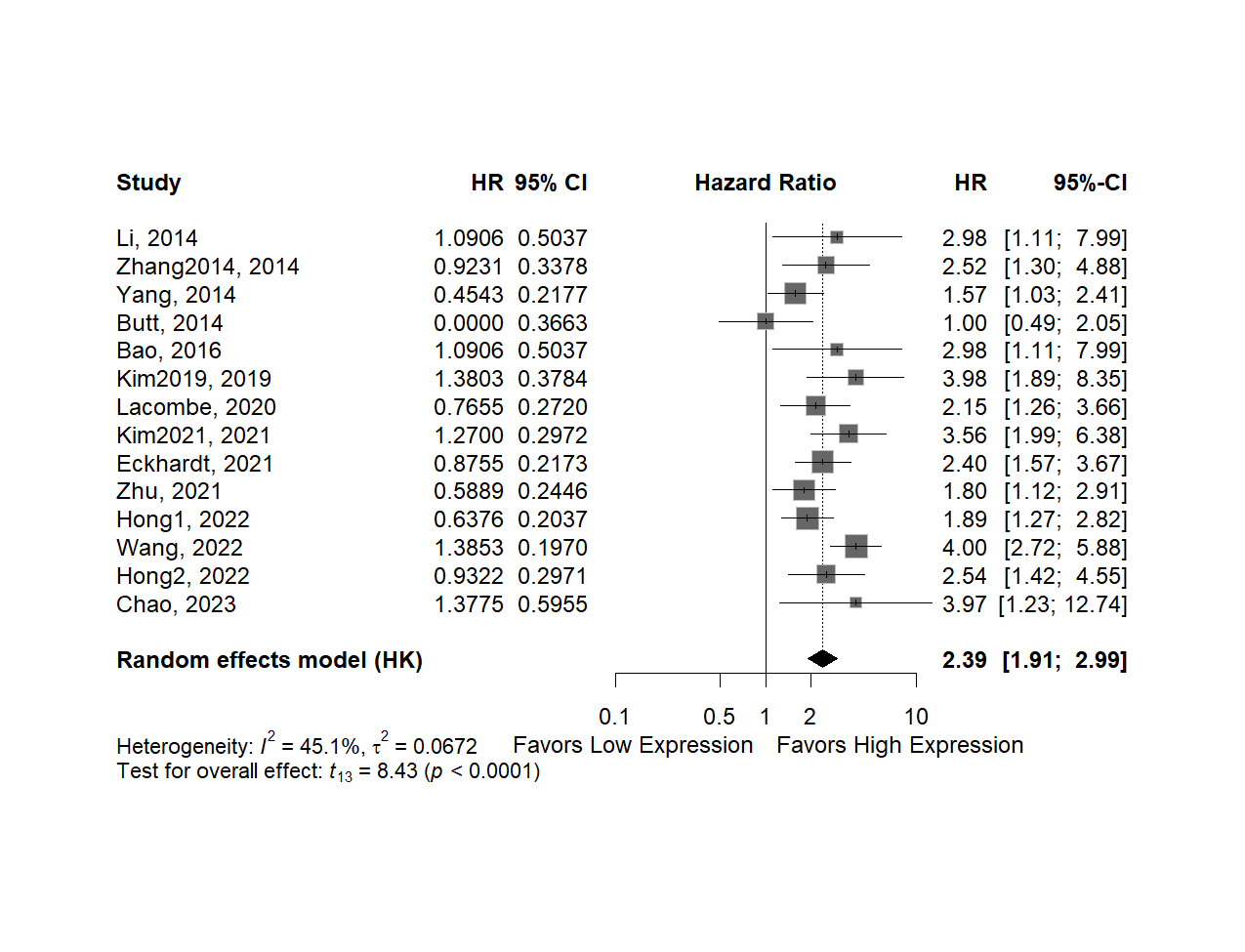

Supplement: Supplementary file 3 — Supplementary Material 3: Figure 3. Sensitivity Analysis Excluding Studies Without Cut-off Values. [file 12885_2025_14633_MOESM3_ESM.png]
