# Supplementary material for: Evaluating cholesterol de novo synthesis biomarkers: a systematic review and meta-analysis of cancer prognosis and clinical outcomes
Source: BMC Cancer. 2025 Jul 24;25:1208. doi: 10.1186/s12885-025-14633-8 (PMC12291504; doi:10.1186/s12885-025-14633-8)
Supplement: Supplementary file 5 — Supplementary Material 5: Table 2. AMSTAR 2 Assessment of Methodological Rigor. Table 3. Comprehensive Assessment of Evidence. Table 4. PRISMA Checklists. [file 12885_2025_14633_MOESM5_ESM.docx]

**Supplementary Table 2. AMSTAR 2 Assessment of Methodological Rigor**

| Item | Description | Rating |
| --- | --- | --- |
| 1 | Research questions and inclusion criteria included | Yes |
| 2* | Protocol registered before review commencement | No |
| 3* | Comprehensive literature search strategy used | Yes |
| 4 | Search strategy justified | Yes |
| 5 | Study selection in duplicate | Yes |
| 6 | Data extraction in duplicate | Yes |
| 7 | Excluded studies listed and justified | Partly yes† |
| 8* | Included studies described in detail | Yes |
| 9* | Risk of bias assessed appropriately | Yes |
| 10* | Funding sources reported for included studies | Yes |
| 11* | Appropriate meta-analytical methods used | Yes |
| 12 | Impact of risk of bias considered in synthesis | Yes |
| 13* | Risk of bias addressed in conclusions | Yes |
| 14 | Heterogeneity explained | Yes |
| 15* | Publication bias assessed | Yes |
| 16 | Conflicts of interest declared | Yes |
| Overall Rating: High |  |  |
| *Critical domains; †Adapted to PRISMA flowchart without full exclusion list | | |

**Supplementary Table 3**: Comprehensive Assessment of Evidence Quality (outcome-specific certainty)

| Outcome | GRADE Certainty | Evidence summary by outcome | AMSTAR 2 Rating |
| --- | --- | --- | --- |
| OS | Low | Moderate heterogeneity (I²=45%) but consistent results; low risk of bias (all studies: NOS ≥6, IHC score ≥15/22) | High |
| DFS | Low | High heterogeneity (I²=65%) offset by robust sensitivity analyses; low bias risk | High |
| RFS | Very Low | Serious inconsistency (I²=89%), imprecision (wide CI: 0.28–3.24), unstable subgroup effects | High |

**Supplementary Table 4:** PRISMA Checklist Item

**PRISMA Checklist Item**

| Section and Topic | Item # | Checklist Item | Location Where Item is Reported |
| --- | --- | --- | --- |
| TITLE | 1 | The report was identified as a systematic review and meta-analysis. | Title, Abstract |
| ABSTRACT | 2 | A structured abstract was provided, including background, methods, results, and conclusions. | Abstract |
| INTRODUCTION | 3 | The rationale for the review was described in the context of existing knowledge. | Background |
| Objectives | 4 | The objectives of the review were explicitly stated. | Abstract, Background |
| METHODS | 5 | The inclusion and exclusion criteria for the review and how studies were grouped for the syntheses were specified. | Methods: Inclusion/Exclusion |
| Information sources | 6 | All databases and sources searched to identify studies were specified, along with the date of the last search. | Methods: Search Strategy |
| Search strategy | 7 | The full search strategies for all databases, including filters and limits used, were presented. | Methods: Search Strategy |
| Selection process | 8 | The methods used to decide whether a study met the inclusion criteria were specified, including the number of reviewers and their independence. | Methods: Data Extraction |
| Data collection process | 9 | The methods used to collect data from reports were specified, including the number of reviewers, their independence, and how discrepancies were resolved. | Methods: Data Extraction |
| Data items | 10a | All outcomes for which data were sought were listed and defined. | Methods: Outcomes |
| Data items | 10b | All other variables for which data were sought were listed and defined; assumptions about missing or unclear information were described. | Methods: Variables |
| Study risk of bias assessment | 11 | The methods used to assess risk of bias in the included studies were specified, including the tools used and the number of reviewers. | Methods: Quality Assessment |
| Effect measures | 12 | For each outcome, the effect measures used in the synthesis or presentation of results were specified. | Methods: Statistical Analysis |
| Synthesis methods | 13a | The processes used to decide which studies were eligible for each synthesis were described. | Methods: Inclusion/Exclusion |
| Synthesis methods | 13b | Any methods required to prepare the data for presentation or synthesis, such as handling of missing summary statistics, were described. | Methods: Data Extraction |
| Synthesis methods | 13c | The methods used to tabulate or visually display results of individual studies and syntheses were described. | Results: Table 1, Figures |
| Synthesis methods | 13d | The methods used to synthesize results and the rationale for the choice were described, including meta-analysis models and heterogeneity assessment. | Methods: Statistical Analysis |
| Synthesis methods | 13e | Any methods used to explore possible causes of heterogeneity among study results were described (e.g., subgroup analysis). | Methods: Statistical Analysis, Results: Subgroup |
| Synthesis methods | 13f | Any sensitivity analyses conducted to assess robustness of the synthesized results were described. | Methods: Statistical Analysis, Results: Sensitivity |
| Reporting bias assessment | 14 | The methods used to assess risk of bias due to missing results (reporting bias) were described. | Methods: Publication Bias |
| Certainty assessment | 15 | The methods used to assess certainty (or confidence) in the body of evidence for an outcome were described. | Methods: GRADE, Results: Certainty |
| RESULTS | 16a | The results of the search and selection process, including the number of records identified, screened, and included, were described (with a flow diagram). | Results: Search Results, Fig. 1 |
| Study selection | 16b | Studies that appeared to meet inclusion criteria but were excluded were cited, with reasons for exclusion. | Results: Search Results |
| Study characteristics | 17 | Each included study was cited and its characteristics were presented. | Results: Table 1. |
| Risk of bias in studies | 18 | Assessments of risk of bias for each included study were presented. | Results: Supplementary Table 1 |
| Results of individual studies | 19 | For all outcomes, summary statistics for each group and effect estimates with precision were presented, using structured tables or plots. | Results |
| Results of syntheses | 20a | For each synthesis, the characteristics and risk of bias among contributing studies were summarized. | Results: Quality Assessment |
| Results of syntheses | 20b | Results of all statistical syntheses conducted were presented, including summary estimates, precision, and heterogeneity measures. | Results |
| Results of syntheses | 20c | Results of all investigations of possible causes of heterogeneity among study results were presented. | Results: Subgroup Analysis |
| Results of syntheses | 20d | Results of all sensitivity analyses conducted to assess the robustness of the synthesized results were presented. | Results: Sensitivity Analysis |
| Reporting biases | 21 | Assessments of risk of bias due to missing results (reporting biases) for each synthesis assessed were presented. | Results: Publication Bias |
| Certainty of evidence | 22 | Assessments of certainty (or confidence) in the body of evidence for each outcome assessed were presented. | Results: Certainty Assessment |
| DISCUSSION | 23a | A general interpretation of the results in the context of other evidence was provided. | Discussion |
| Discussion | 23b | Limitations of the evidence included in the review were discussed. | Discussion |
| Discussion | 23c | Limitations of the review processes used were discussed. | Discussion |
| Discussion | 23d | Implications of the results for practice, policy, and future research were discussed. | Discussion |
| OTHER INFORMATION | 24a | Registration information for the review was provided, or it was stated that the review was not registered. | Methods: Not registered |
| Registration and protocol | 24b | Whether a review protocol was prepared and where it can be accessed was indicated, or it was stated that no protocol was prepared. | Supplementary file: prepared |
| Registration and protocol | 24c | Any amendments to information provided at registration or in the protocol were described and explained. | Methods: Not applicable |
| Support | 25 | Sources of financial or non-financial support for the review and the role of funders or sponsors were described. | Not applicable |
| Competing interests | 26 | Competing interests of review authors were declared. | Declarations |
| Availability of data, code, materials | 27 | Which of the following are publicly available and where they can be found: template data collection forms; data extracted from included studies; data used for all analyses; analytic code; any other materials used in the review were reported. | Declarations |

The abbreviations;OS (Overall Survival), DFS (Disease-Free Survival), RFS (Recurrence-Free Survival), GRADE (Grading of Recommendations, Assessment, Development, and Evaluation), AMSTAR 2 (A MeaSurement Tool to Assess systematic Reviews 2), I² (I-squared statistic for heterogeneity), NOS (Newcastle-Ottawa Scale), and IHC (Immunohistochemistry).
