# Supplementary material for: Evaluating cholesterol de novo synthesis biomarkers: a systematic review and meta-analysis of cancer prognosis and clinical outcomes
Source: BMC Cancer. 2025 Jul 24;25:1208. doi: 10.1186/s12885-025-14633-8 (PMC12291504; doi:10.1186/s12885-025-14633-8)
Supplement: Supplementary file 6 — Supplementary Material 6. [file 12885_2025_14633_MOESM6_ESM.docx]

**Study Protocol**

**Title Page**

**Evaluating Cholesterol** **De Novo Synthesis Biomarkers: A Systematic Review and Meta-Analysis of Cancer Prognosis and Clinical Outcomes**

**Authors and Affiliations:**

**Eman Taha Osman Ali,**

**Department of Histopathology and Cytology, University of Khartoum,** **Khartoum, Sudan**

**Nouh Saad Mohamed,**

**Sirius Training and Research Centre, Khartoum, Sudan**

**Emmanuel Edwar Siddig**

Faculty of Medical Laboratory sciences, University of Khartoum, Khartoum, Sudan

**Mai Abdulrahman Mohammed Masr**

Molecular Biology Department, Faculty of Zoology, University of Khartoum, Khartoum, Sudan

**Corresponding Author:**

**Eman Taha Osman Ali**

**Email: 211mgg02@ewhain.net**

**ORCID:** <https://orcid.org/0000-0001-6843-3361>**.**

1**. Background and Rationale**

Cholesterol de novo synthesis is increasingly recognized as a hallmark of cancer metabolism, influencing proliferation, survival, and drug resistance. Multiple markers involved in this pathway—such as SREBP1, SREBP2, HMGCR, SOAT1, and SQLE—have been studied as prognostic indicators in solid tumors. However, their predictive value remains inconsistent across individual studies. This protocol outlines a systematic review and meta-analysis aimed at quantitatively evaluating the prognostic and clinicopathological significance of cholesterol synthesis markers in human cancers.

**2. Objectives**

**Primary Objective:**

To assess the association between the expression of cholesterol synthesis markers and overall survival (OS), disease-free survival (DFS), and recurrence-free survival (RFS) in patients with solid tumors.

**Secondary Objective:**

To determine the relationship between these markers and clinicopathological features, including tumor size, differentiation, lymph node status, and age.

**3. Study Design**

This is a systematic review and meta-analysis conducted according to the PRISMA 2020 guidelines. The review protocol is designed in alignment with Cochrane recommendations and the AMSTAR 2 tool for quality appraisal.

**4. Eligibility Criteria**

Inclusion Criteria:

Studies assessing cholesterol synthesis markers (e.g., SREBP1/2, SOAT1, SQLE, HMGCR) in solid human tumors.

Studies reporting hazard ratios (HRs) for OS, DFS, or RFS.

Original research articles with full-text available in English.

Immunohistochemistry (IHC), RT-PCR, or Western blotting used for marker detection.

**Exclusion Criteria:**

Non-solid tumor studies (e.g., hematologic malignancies).

Conference abstracts, editorials, reviews, or case reports.

Studies lacking sufficient data to compute HRs or ORs.

**5. Information Sources and Search Strategy**

Databases:

PubMed

EMBASE

Cochrane Library

Search Terms:

(SREBP1 OR SREBP2 OR SOAT1 OR HMGCR OR SQLE OR "Sterol Regulatory Element Binding Proteins" OR "cholesterol synthesis") AND (cancer OR tumor OR carcinoma OR neoplasm) AND (prognosis OR survival OR outcome)

Language: English only.

6**. Study Selection Process**

Duplicates removed using EndNote.

Two reviewers independently screen titles and abstracts.

Full texts of potentially relevant articles are assessed for eligibility.

Disagreements are resolved by consensus or a third reviewer.

**7. Data Extraction**

First author, year, country

Study design

Cancer type and sample size

Marker assessed and detection method

HRs and ORs with 95% CIs

Cut-off values used

Clinicopathological parameters

Follow-up duration

8. **Methodological Quality Assessment**

Each study will be scored using a modified 18-point methodological scoring tool (as outlined in your previous message). Criteria include:

Study description (age, gender, histology, recruitment period)

Attrition and follow-up

IHC methodology detail

Scoring techniques

Confounding factor control

Statistical transparency (HRs, p-values, KM data)

Scores will inform sensitivity analyses and contribute to GRADE’s "risk of bias" assessment.

9. Risk of Bias Assessment

The methodological scoring system above will serve as a semi-quantitative risk of bias tool.

GRADE will be applied at the outcome level to assess:

10. **Data Synthesis and Statistical Analysis**

Software:

All analyses will be conducted using R version 3.2.2 and the “meta” package.

**Analyses:**

Pooled HRs and ORs using inverse variance methods.

Random-effects model applied when I² > 25%; fixed-effects model for I² = 0.

Subgroup analyses based on cancer type, marker type, and cut-off reporting.

Sensitivity analysis by excluding low-quality studies or outliers.

**Heterogeneity:**

Quantified using I² statistic and p-value.

Low: I² < 25%, Moderate: 25–75%, High: >75%

**Publication Bias:**

Evaluated using Egger’s test.

11. Ethics and Dissemination

As this study uses previously published data, no ethical approval is required. Results will be submitted to BMC Cancer and disseminated via scientific open-access platforms.

12. **Registration**

This protocol was not pre-registered.

13. **Amendments**

Any future protocol changes will be clearly documented and explained in the final manuscript.
